# DeepECA: an end-to-end learning framework for protein contact prediction from a multiple sequence alignment

**DOI:** 10.1186/s12859-019-3190-x

**Published:** 2020-01-09

**Authors:** Hiroyuki Fukuda, Kentaro Tomii

**Affiliations:** 1 Department of Computational Biology and Medical Sciences, Graduate School of Frontier Sciences, The University of Tokyo, 5-1-5 Kashiwanoha, Kashiwa-shi, Chiba-ken, 277-8562 Japan; 2 Artificial Intelligence Research Center (AIRC), Biotechnology Research Institute for Drug Discovery, Real World Big-Data Computation Open Innovation Laboratory (RWBC-OIL), National Institute of Advanced Industrial Science and Technology (AIST), 2-4-7 Aomi, Koto-ku, Tokyo, 135-0064 Japan

**Keywords:** Contact prediction, Convolutional neural network, Deep learning, Multiple sequence alignment, Protein, Secondary structure prediction

## Abstract

**Background:**

Recently developed methods of protein contact prediction, a crucially important step for protein structure prediction, depend heavily on deep neural networks (DNNs) and multiple sequence alignments (MSAs) of target proteins. Protein sequences are accumulating to an increasing degree such that abundant sequences to construct an MSA of a target protein are readily obtainable. Nevertheless, many cases present different ends of the number of sequences that can be included in an MSA used for contact prediction. The abundant sequences might degrade prediction results, but opportunities remain for a limited number of sequences to construct an MSA. To resolve these persistent issues, we strove to develop a novel framework using DNNs in an end-to-end manner for contact prediction.

**Results:**

We developed neural network models to improve precision of both deep and shallow MSAs. Results show that higher prediction accuracy was achieved by assigning weights to sequences in a deep MSA. Moreover, for shallow MSAs, adding a few sequential features was useful to increase the prediction accuracy of long-range contacts in our model. Based on these models, we expanded our model to a multi-task model to achieve higher accuracy by incorporating predictions of secondary structures and solvent-accessible surface areas. Moreover, we demonstrated that ensemble averaging of our models can raise accuracy. Using past CASP target protein domains, we tested our models and demonstrated that our final model is superior to or equivalent to existing meta-predictors.

**Conclusions:**

The end-to-end learning framework we built can use information derived from either deep or shallow MSAs for contact prediction. Recently, an increasing number of protein sequences have become accessible, including metagenomic sequences, which might degrade contact prediction results. Under such circumstances, our model can provide a means to reduce noise automatically. According to results of tertiary structure prediction based on contacts and secondary structures predicted by our model, more accurate three-dimensional models of a target protein are obtainable than those from existing ECA methods, starting from its MSA. DeepECA is available from https://github.com/tomiilab/DeepECA.

## Background

Many methods have been developed for protein contact prediction, a crucially important step for protein structure prediction [1–19]. In the earlier stages of contact prediction history, most successful prediction methods were based on evolutionary coupling analysis (ECA) of large multiple sequence alignments (MSAs) of homologous sequences. In evolutionary processes, pairs of residues that are mutually proximate in the tertiary structure tend to co-evolve to maintain their structure. For instance, when one becomes larger, the other becomes smaller. Alternatively, when one becomes a positively charged residue, the other becomes a negatively charged residue.

Usually, evolutionary information includes noise because of indirect correlation between residues (A and B) when residues (A and C) and residues (B and C) are directly correlated. True correlation must be distinguished from such noise. Many challenges have been undertaken to do so. The methods used to address them can be categorized into two groups: Graphical Lasso and pseudo-likelihood maximization. Friedman et al. developed Graphical Lasso, a graph structure estimation method, in 2008 [20]. It can estimate the graph structure from a covariance matrix using likelihood estimation of a precision matrix with L1 regularization. A well-known program that applies Graphical Lasso to contact prediction problems is PSICOV [4]. A pseudo-likelihood method is used for an approximation method for probabilistic models, such as a Potts model, to estimate interaction strength between residues. It is usually difficult to calculate the marginal probability exactly. For that reason, such an approximation method is often used. Major programs using this method are EVFold [5], plmDCA [11], GREMLIN [7], and CCMpred [13].

After these extensive studies of ECA, meta-predictors emerged. The methods achieve protein contact prediction using the ECA method results as input features. MetaPSICOV [14], a well-known supervised method, uses outputs of PSICOV, CCMpred, and FreeContact [12] as input features and uses many other features such as secondary structure probability, solvent accessibility, and Shannon entropy. Using 672 features in this way, MetaPSICOV improved prediction accuracy much more than a single ECA method can. Subsequently, Wang et al. [19] proposed a method based on an ultra-deep residual neural network and achieved much higher accuracy than had ever been attained previously. The recently reported DeepCov [21], which is a conceptually similar method to ours uses a covariance matrix calculated from MSA for input features for DNN. For the 13th Community Wide Experiment on the Critical Assessment of Techniques for Protein Structure Prediction (CASP13), several groups used a deep neural network (DNN) for contact prediction. Among them, ResPRE [22] used a precision matrix instead of a covariance matrix and DeepMetaPSICOV [23] which combined the covariance-based method, DeepCov and features from MetaPSICOV.

**Fig. 1:**
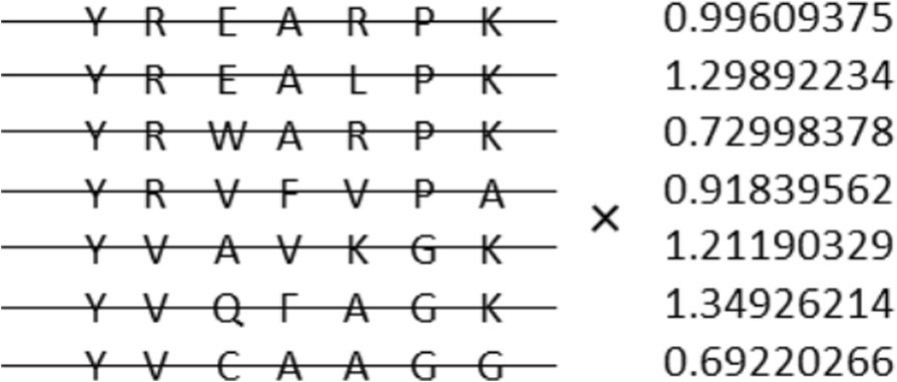
Schematic representation of weighted MSA: The left panel shows a part of the MSA. The right panel shows weight values for each sequence in the MSA

Nevertheless, despite recent success achieved using these methods, most of them do not predict contacts from MSA directly. None has any means of optimizing the input MSAs. Some room for improvement remains for contact prediction pipeline optimization. As presented herein, we describe a novel approach to contact prediction that can extract correlation information, and which can predict contacts directly from MSA using a DNN in an end-to-end manner. Using DNN, one can outperform existing ECA methods, MetaPSICOV, DeepCov, ResPRE and DeepMetaPSICOV, and obtain comparable accuracy to that of RaptorX-Contact [19] using no other additional input feature such as secondary structures. Furthermore, our DNN-based method can provide a means of optimizing the input MSAs in a supervised manner. The weight of each sequence in MSA is parameterized (Fig. 1). It can be optimized through DNN to eliminate noise sequences in MSA automatically. In this model, we expect that more important sequences have greater weights and that less-important sequences have less weight after optimization. Today, a growing number of protein sequences are obtainable so that not all sequences in MSA necessarily have the same contacts. These sequences can introduce noise that affects contact prediction. In addition, Fox et al. [24] reported that the contact prediction accuracy depends on the MSA accuracy. Motivated by those findings, we attempt to weight the sequences of MSA correctly. We also report that adding features and ensemble averaging can raise accuracy considerably and that high accuracy of secondary structures prediction can be achieved with our contact model using multi-task learning. Our experiments demonstrate that addition of a few features and the use of ensemble averaging are effective means of raising accuracy. High accuracy of secondary structures and accessible surface area prediction can be achieved using our contact model with multi-task learning. This result of multi-task learning suggests that contact information includes secondary structure and accessible surface area information. It can help to raise the accuracy of these predictions. Finally, we build a tertiary structure solely from predicted contacts and predicted secondary structures and retrieve a TMscore [25] greater than 0.5 for 50 out of 105 (48%) CASP11 domains and 18 out of 55 (33%) CASP12 domains.

## Results

### Effects of weighting sequences in an MSA

**Fig. 2:**
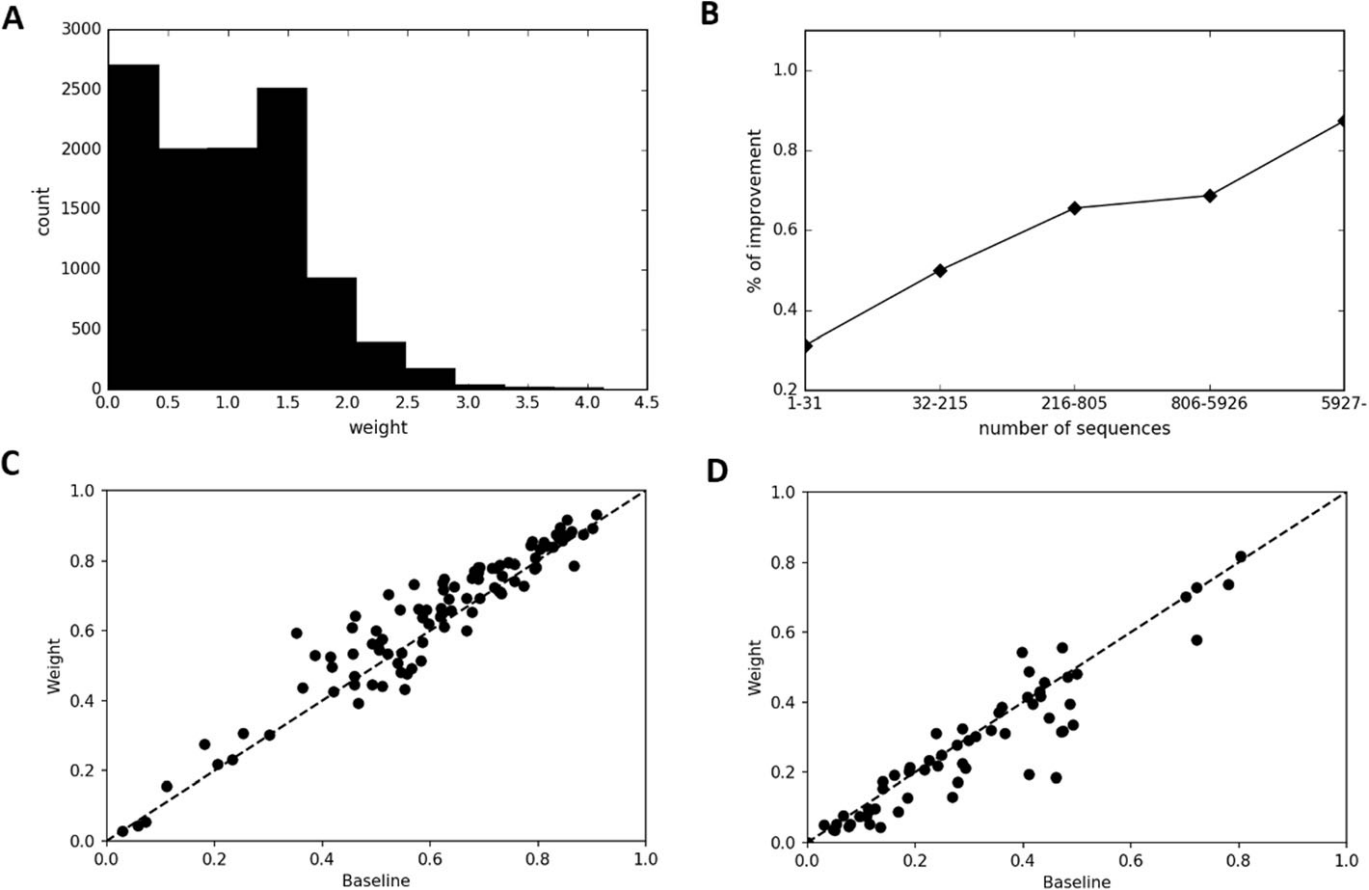
**a** One example of weight distribution in the sequences of one MSA for T0843 on the CASP11 dataset. **b** Accuracy improvement depends on the number of sequences in an MSA. We divided 160 protein domains into five bins according to their lengths. The numbers of proteins in the bins are equal (i.e., 32 protein domains in each bin). **c** Baseline Model top *L* accuracy shown against the Weighted MSA Model when we have over 200 homologous sequences and **d** with fewer than 200 homologous sequences

Here, we demonstrate that weighting of sequences in an MSA can boost prediction accuracy. Our network can learn correctly how to weight the MSA sequence. Figure 2a presents the distribution of the weight values of one protein. Results show that some values were nearly zero, which indicates that some noise sequences were present in the original MSA.

To investigate the result further, we calculate the prediction accuracy dependence on the number of sequences in MSA using 160 protein domains of the CASP11 and CASP12 datasets. For these assessments, we select the results of Long top *L* prediction as a measure of accuracy because this area has the greatest number of predictions and because the standard deviation is smallest. Figure 2b shows that we can improve the prediction accuracy of more than 70% of targets when we have more than 200 sequences, but we cannot improve it when we have only a few sequences. The percentage of improvement is the number of improved proteins divided by the total number of proteins in a bin. This result demonstrates that the network can remove noise sequences when MSA has numerous homologous sequences. Figures 2c and d show an accuracy comparison between our Baseline Model and Weighted MSA Model (about our models, see Method), which also supports our result.

Another approach to test our models is to increase the noise sequences in MSA and testing of the prediction accuracy robustness. We use HHblits and set *E*-values 1 and 3 and eliminate the “-cov” option to produce noisy MSAs and to predict contacts using these noisy MSAs as input. Table 1 presents the results. Because of the increasing noise, the prediction accuracy of Baseline Model is decreasing but that of Weighted MSA Model largely retains its accuracy. This result also indicates that our Weighted MSA Model can eliminate noise sequences.

**Table 1:**
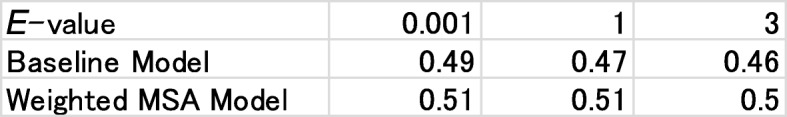
Top *L* Contact Prediction Accuracy on the CASP11 dataset against HHblits e-values

In the experiments conducted on the CASP11 and CASP12 datasets, but not in all prediction categories, we can improve accuracy using the Weighted MSA Model. To assess the effects of weighting sequences further, we compare the accuracies of the Baseline Model and the Weighted MSA Model on one of our five validation datasets. The best epochs of each model are determined by the average loss of the validation set. Using these epochs, the accuracies of the models are calculated. Table 2 shows that the accuracies of the Weighted MSA Model are higher than those of the Baseline Model at every distance and prediction count. These differences were inferred as significant from Student’s *t*-test results.

**Table 2:**
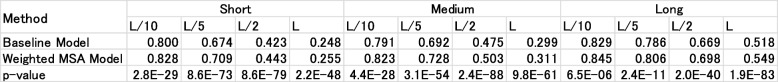
Accuracy comparison between the Baseline Model and the Weighted MSA Model tested on the validation dataset and the *p*-value of Student’s *t*-test

To investigate the extent to which each feature (gap ratio, sequence identity and sequence identity with a consensus sequence) contributes to improvement of accuracy, we train the Weighted MSA Model without each feature and their average values. Furthermore, we compare the prediction accuracies for the validation dataset. The results are shown as “Drop Consensus”, “Drop Identity”, and “Drop Gap Ratio” models in Table 3a. Prediction accuracies of these feature-dropped models are between those of the Baseline Model and the Weighted MSA Model. The accuracy becomes lowest when we drop sequence identity with a consensus sequence and its average value, which means that the contribution of this feature to the accuracy is the highest among three features. The contribution of the gap ratio is the smallest, but a slight contribution is observed in Medium *L*/5 and Long *L*/5 categories.

In the paper describing PSICOV, another method to weight sequences in MSA was introduced before ours. It weights sequences in an MSA using several redundant sequences in the MSA to eliminate redundancy. However, it is not optimized in an end-to-end manner. To compare the accuracy of these two weighting methods, we calculate the weight values of PSICOV separately and apply them to our Baseline Model. The result is presented as the “Baseline+PSICOV” model in Table 3 (B). In this experiment using our weighting method, the Weighted MSA Model is equivalent to or better than “Baseline+PSICOV” model at every distance and prediction count.

**Table 3:**
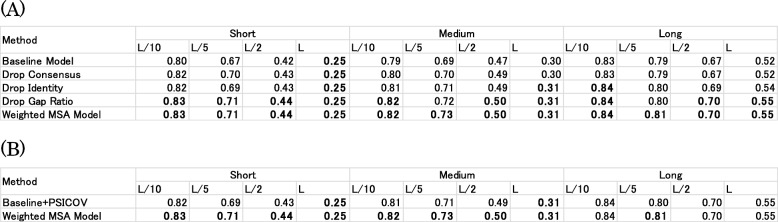
Accuracy comparisons of (**a**) the dropped feature models and (**b**) the weighing method of PSICOV against the Weighted MSA Model tested on the validation dataset. Bold typeface characters show the highest accuracy in the columns

**Fig. 3:**
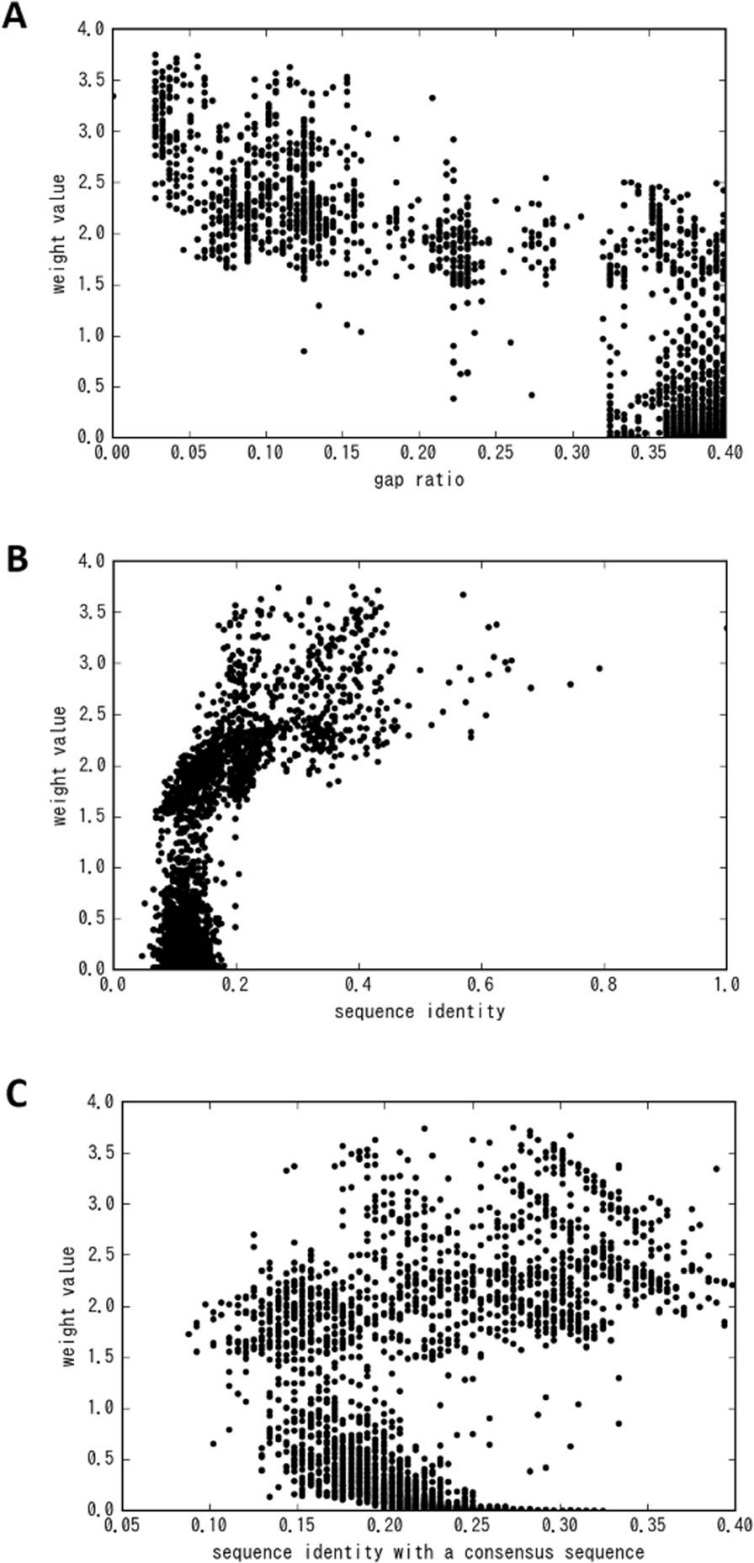
Distributions of weight values of (**a**) the gap ratio, (**b**) sequence identity and (**c**) identity with a consensus sequence. Each dot represents a sequence in the MSA of 1EEJ

Finally, we present distributions of sequence weights calculated using the Weighted MSA Model for a protein chain from the validation dataset. The calculated weights are shown respectively against the gap ratio, sequence identity, and sequence identity with a consensus sequence (Fig. 3). As shown in Figs. 3 and S1, dependencies of sequence weights against their gap ratio and sequence identity can be observed to some extent in some cases. However, such dependencies are not always evident. As described above, sequence identity with a consensus sequence and its average value have the highest contribution to our model. The relations between weights and this feature are complicated. At least, these are not linear dependencies (perhaps because we use DNN to weight the sequences). Other examples of relations between weights and features are shown in Additional file 1: Figure S1. These plots show that these relations vary depending on proteins and their MSAs.

### Effects of adding features

**Fig. 4:**
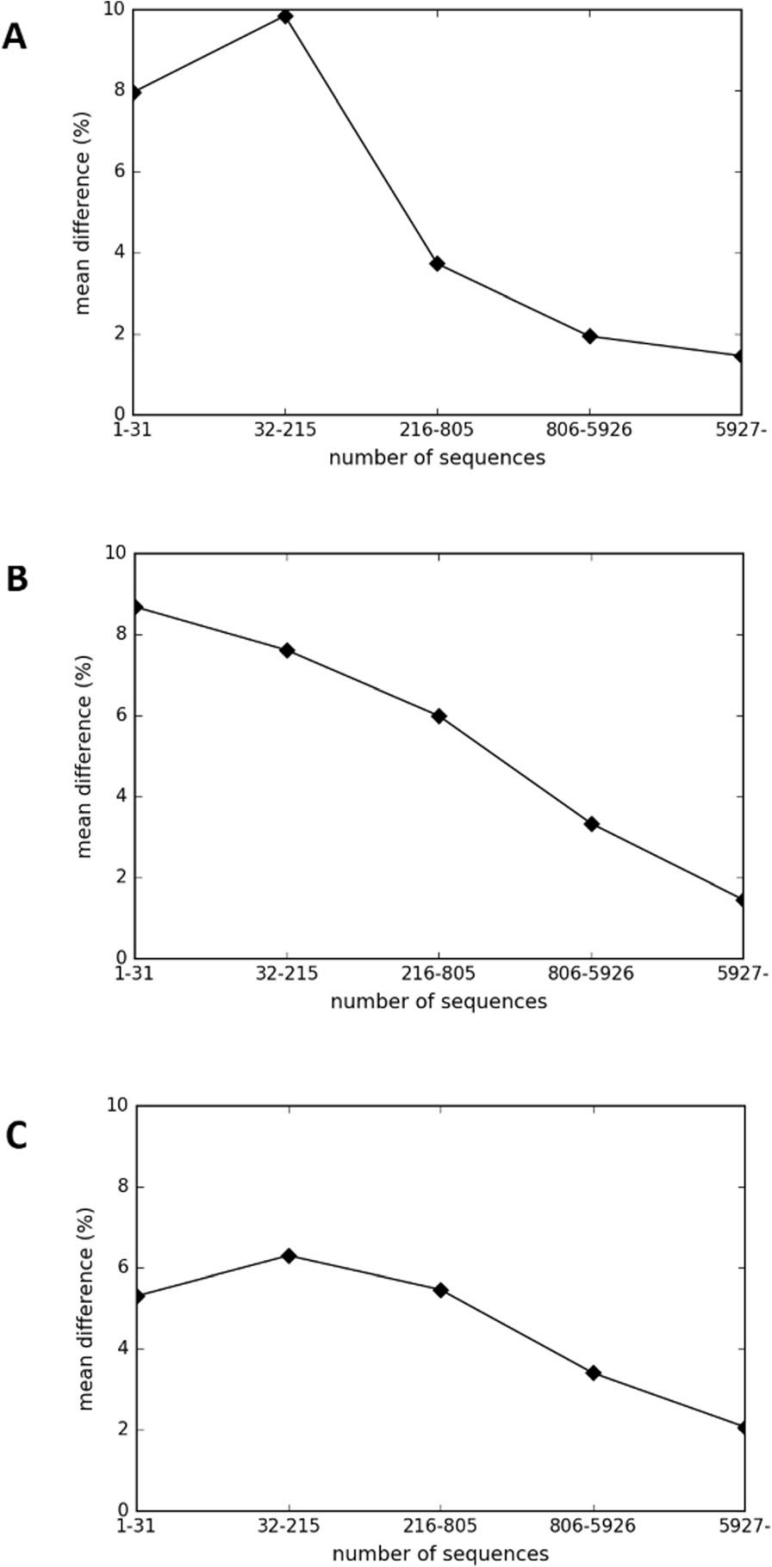
Accuracy improvement depends on the number of sequences in an MSA. The mean differences of prediction accuracy, between the Feature Added model and the Weighted MSA Model, against the number of sequences in an MSA, are shown for (**a**) top *L*/5, (**b**) top *L*/2, and (**c**) top *L* contacts of prediction at long range. The number of proteins in each bin is equal (i.e., 32 protein domains in each bin)

In our experiments, adding a few sequential features was useful for increasing the prediction accuracy in cases with shallow MSAs. Results showed that the Feature Added Model can produce considerable accuracy gains of prediction at long range for the CASP11 and CASP12 datasets (Fig. 4). Although DNN can find useful features automatically, handmade feature engineering is still effective in our experiments. For this experiment, we added five features, as described in Method.

### Effects of multi-task learning

Presumably, a predicted contact map includes secondary structure information. Based on this assumption, we tried to use multi-task learning to predict contacts and secondary structures simultaneously. We examined three state secondary structure prediction. Table 4 presents the results. Our method outperformed existing methods such as RaptorX-Property [26] and SCRATCH-1D [27] in terms of prediction accuracy. This result demonstrates that our 2D feature maps are a good representation of secondary structure prediction. It also demonstrates that we can extract useful information from these feature maps through multi-task learning. In our experiments, convergence of the secondary structure prediction differed from that of contact prediction. We use the best epoch of each. SCRATCH-1D uses structural data from PDB to predict secondary structures. The time stamp of the structural data is June 2015, which is after the CASP11 experiment. This might explain why SCRATCH-1D obtains better results with the CASP11 dataset than the results obtained using the CASP12 dataset.

**Table 4:**
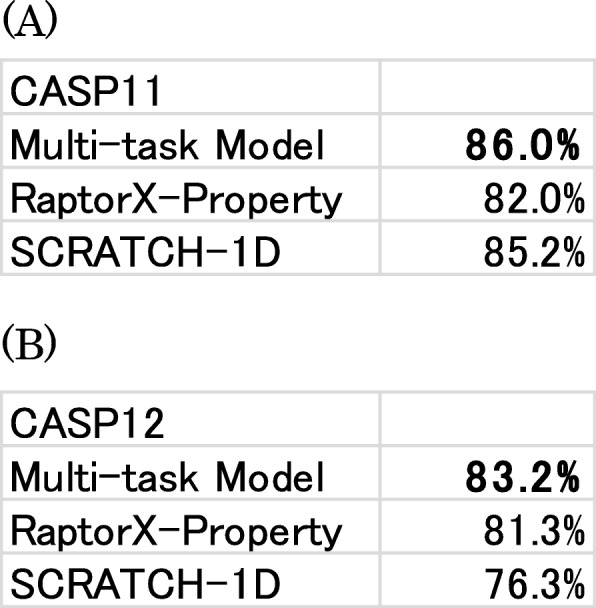
Secondary structure prediction accuracy on the (**a**) CASP11 and (**b**) CASP12 datasets. Bold typeface characters show the highest accuracy in the column

To investigate these results further, the recall and precision of each predicted secondary structure class on the CASP11 and CASP12 datasets are calculated and are presented in Table 5. The model shows especially good results for precision of sheet prediction on both the CASP11 and CASP12 datasets. Although SCRATCH-1D shows better results for the recall of helix and sheet prediction and precision of coil prediction on the CASP11 dataset because of the structural data used in SCRATCH-1D, our model outperforms the other two methods in almost all classes on the CASP12 dataset.

**Table 5:**
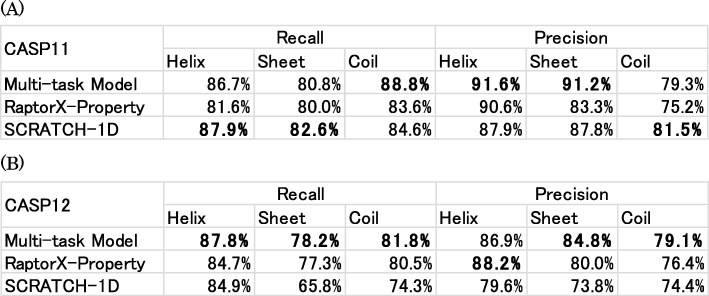
Recall and precision of secondary structure components on the (**a**) CASP11 and (**b**) CASP12 datasets. Bold typeface characters show the highest accuracy in the column

We also compared the prediction results of accessible surface area with those obtained using two other methods. Our model, which is a regression model, outputs the predicted accessible surface area as a real number. However, RaptorX-Property is a classification model that outputs the relative solvent accessibility in three states: B, Buried; M, Medium; and E, Exposed. (10 and 40% are the thresholds). Furthermore, SCRATCH-1D outputs relative solvent accessibility in 20 classes (0–95% in 5% increments). To compare these three results, the results of our models and SCRATCH-1D are converted to three state prediction, similarly to RaptorX-Property. As in secondary structure prediction, our model can obtain the highest accuracies among these three methods (Table 6).

**Table 6:**
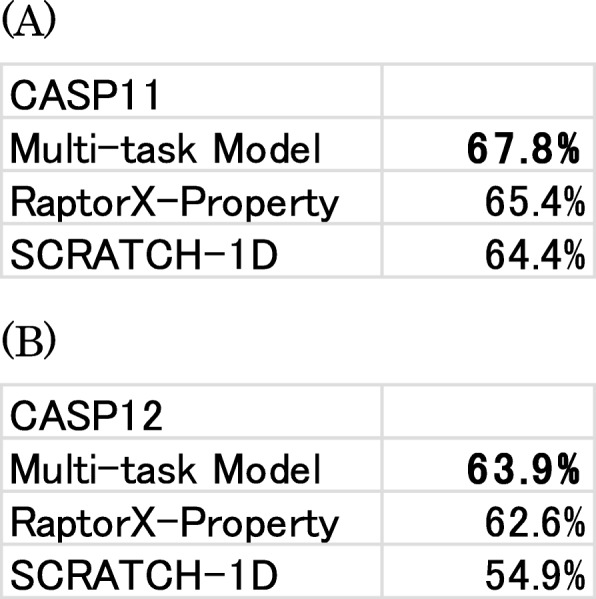
Accessible surface area prediction accuracy on the (**a**) CASP11 and (**b**) CASP12 datasets. Bold typeface characters show the highest accuracy in the columns

Finally, we analyze what types of contacts (e.g. helix–helix, helix–sheet and sheet–sheet) are better predicted with the Feature Added Model and the Multi-task Model. Table 7 shows the results. On both the CASP11 and CASP12 dataset, recalls of the Multi-task Model are equivalent to or higher than those of the Feature Added Model for contacts of all three types rather than a particular type of contact. Regarding precision, the sheet–sheet contact of the Feature Added Model is better than that of the Multi-task Model. The secondary structure types contribute somewhat to the contact prediction accuracy.

**Table 7:**
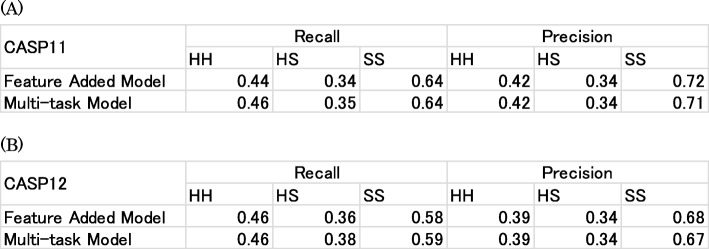
Recall and Precision of three types of contact: helix–helix (HH), helix–sheet (HS), and sheet–sheet (SS) on the (**a**) CASP11 and (**b**) CASP12 datasets

### Effects of ensemble averaging

Regarding the model ensemble, according to the machine learning theory, ensemble methods of some types exist such as bagging, boosting, and stacking. Our ensemble averaging is similar to bagging. It uses bootstrapping samples as training data. However, in our case, we use datasets from cross validation. Generally, ensemble models use weak classifiers such as a decision tree as a base model. We use DNN, which is not regarded as a weak classifier. However, in our experiments, the ensemble model is still effective. Tables 8 and 9 show that ensemble-learning can raise the accuracy considerably for almost all prediction categories, except Medium top *L*/10 prediction on the CASP12 dataset.

**Table 8:**
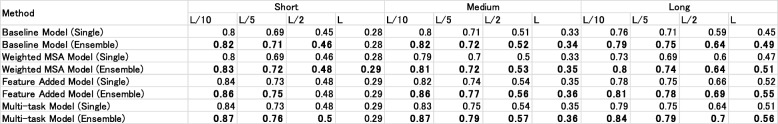
Contact prediction accuracy comparison between single learning and ensemble averaging on the CASP11 dataset. Bold typeface characters show that ensemble averaging can raise the accuracy of this field

**Table 9:**
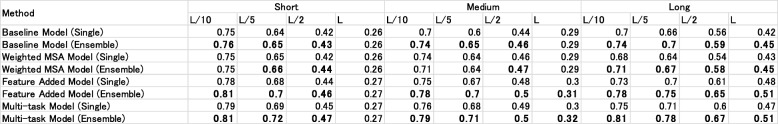
Contact prediction accuracy comparison between single learning and ensemble averaging on the CASP12 dataset. Bold typeface characters signify that ensemble averaging can raise the accuracy of this field

We also investigate how contact prediction accuracy depends on the training datasets in our ensemble averaging. We test 3-, 5-, 7-, and 10-fold and compare the respective degrees of accuracy using a Baseline Model. Generally, it is expected that as the number of folds increases, prediction accuracy is also increasing, but it eventually reaches a plateau because the overlap of data is large and because the model diversity becomes small. Table 10 shows that the 10-fold result yields the highest accuracy at almost all prediction categories. However, the difference is not so large. We use 5-fold to save computational time for all experiments.

**Table 10:**
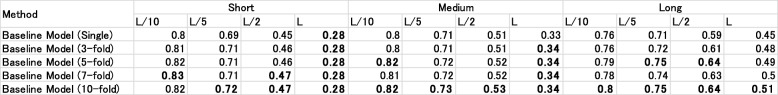
Dependencies of prediction accuracy on the number of folds on the CASP11 dataset. Bold typeface characters show the highest accuracy in the column

### Accuracy comparison for the CASP11 and CASP12 targets

Tables 11 and 12 respectively present the predictive accuracies of five existing methods and our methods. We evaluated our method using the CASP11 and CASP12 datasets. Both the CASP11 and CASP12 datasets yielded similar results. Even our baseline method outperformed existing ECA methods at every distance and prediction count. Additionally, our baseline model outperformed DeepCov, which also takes the covariance matrices as input and which uses DNN. Comparison against other existing models revealed that the Multi-task Model can outperform metaPSICOV, ResPRE, and DeepMetaPSICOV, and that it can obtain comparable results to those of RaptorX-Contact.

**Table 11:**
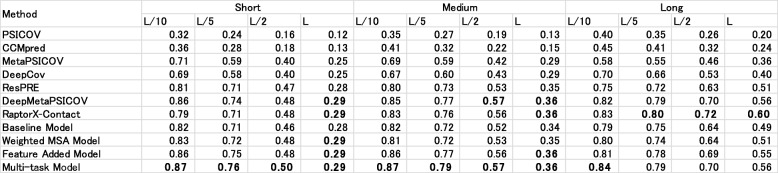
Contact prediction accuracy on the CASP11 dataset. Bold typeface characters show the highest accuracy in the column

**Table 12:**
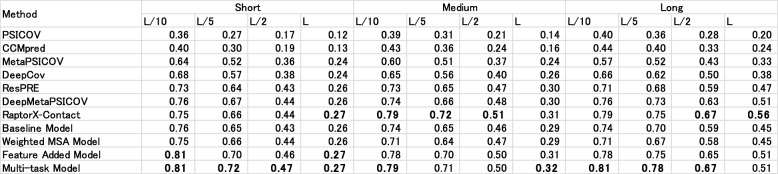
Contact prediction accuracy on the CASP12 dataset. Bold typeface characters show the highest accuracy in the column.

Among our models, results show that Weighted MSA, Feature Added, and Multi-task Models can gradually raise the total accuracy compared with our baseline model, except for Weighted MSA Model in CASP12. The Weighted MSA Model is ineffective in such situations because most CASP12 targets have an insufficient number of homologous sequences in MSA.

### Tertiary structure prediction

**Fig. 5:**
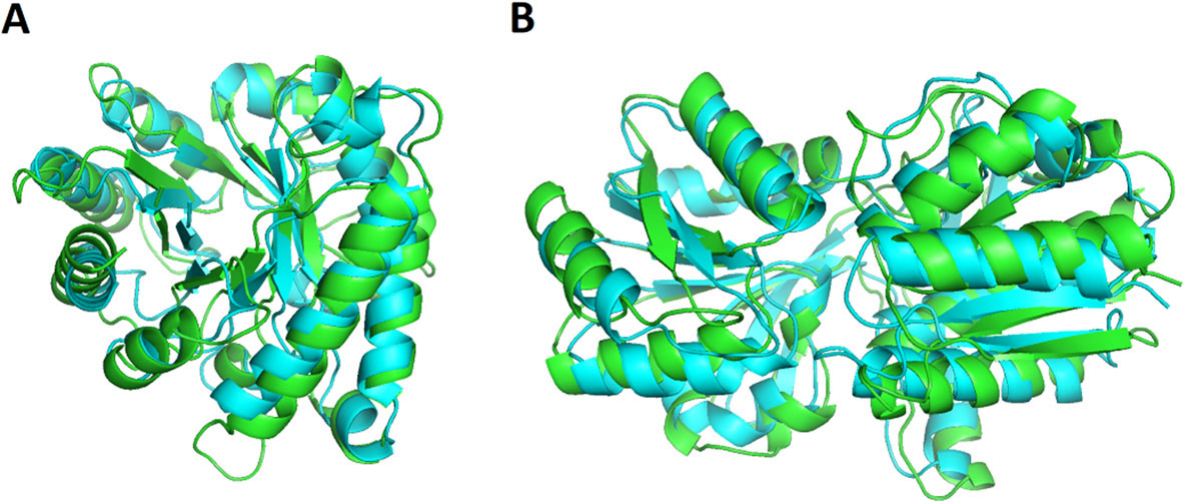
(**a**) Our best predicted model T0811-D1 in CASP11 and (**b**) T0920-D1 in CASP12. Cyan shows the native structure. Green represents our model

From the predicted contacts and secondary structures obtained using our Multi-task Model, we attempt to construct tertiary structures using the CONFOLD script [28]. We measure the quality of predicted structures in terms of the TMscore. The average TMscores are 0.472 (CASP11) and 0.402 (CASP12). We can obtain a TMscore over 0.5 only by MSA information against 50 in 105 (48%) of CASP11 domains and 18 in 55 (33%) of CASP12 domains. Especially when we have more than 0.8 top *L* predicted contact accuracy, the numbers improve to 17 in 22 (77%) of CASP11 domains and 5 in 7 (71%) of CASP 12 domains. Here, we present an example of the best predicted structure T0811-D1 (TMscore 0.818) in CASP11 and T0920-D1 (TMscore 0.848) in CASP12 (Fig. 5). In these domains, the accuracies of top *L* contact predictions are 85.3% (T0811-D1) and 86.3% (T0920-D1).

### Calculation time

In terms of calculation time, our method also exhibits good performance. We compare the calculation time of our method with that of CCMpred, which is the fastest method among existing ECA methods. Table 13 shows that our method takes much less time than the CCMpred with or without GPU, when we used 150 proteins in the PSICOV dataset. Although Graphical Lasso and pseudo-likelihood methods have iterative calculations, neural network methods can calculate the result directly. Results are obtainable in a short time once one has completed network training. Our method is practically useful when huge numbers of contact predictions are necessary.

**Table 13:** Calculation time of CCMpred and our method

	CCMpred (CPU)	CCMpred (GPU)	Our DNN (CPU)	Our DNN (GPU)
Time (min)	585	47	10	2

## Discussion

This report presented a novel approach of end-to-end learning for protein contact prediction. On the CASP11 and CASP12 test proteins, for all precisions (short, medium, and long), we confirmed that our models performed better than any other ECA method. Moreover, we were able to obtain comparable results to those obtained using RaptorX-Contact, a successful prediction method that uses outputs of an ECA method (CCMpred) and additional features as inputs, although we use much simpler features derived from an MSA as inputs. Using our prediction results including secondary structures as inputs of other meta-predictors might engender higher precision.

When extracting correlation information for one residue pair, 21 × 21 correlation scores from 21 × 21 amino acid pairs are obtained. However, these scores are merely averaged in PSICOV. By contrast, our method uses 441 covariance matrices as input features and feeds them to the CNN architecture. This method does not engender loss of information, which is an important benefit of our method compared to PSICOV. Moreover, the CNN architecture can extract useful features from covariance matrices automatically through convolutional operation.

Comparison with existing meta-predictors such as metaPSICOV, DeepMetaPSICOV, and RaptorX-Contact revealed that, although we use only correlation information based on an MSA and use no other feature such a secondary structure as input, all our methods outperformed metaPSICOV. Moreover, the Multi-task Model outperformed DeepMetaPSICOV and yielded comparable results to those obtained using RaptorX-Contact. Our methods show better results for short range prediction than results obtained with RaptorX-Contact.

Using DNN, we can not only raise the accuracy of contact prediction: we also have an opportunity to weight sequences in an MSA in an end-to-end manner. Recently, we have become able to access an increasing number of protein sequences including metagenomic sequences, which can include many noise sequences for contact prediction. In such situations, our method provides a means to eliminate noise sequences automatically and to find relevant ones.

Results of our study demonstrate that adding features and using ensemble averaging can raise accuracy. Furthermore, we demonstrate that we can obtain high prediction accuracy of contact, secondary structure and accessible surface area prediction in one network merely using MSA information. This result illustrates that contact information strongly regulates the secondary structure but that the secondary structure information does not include contact information. Recently, Hanson et al. [29] described that the predicted contact maps improve the accuracy of secondary structure prediction. Our result is consistent with those described in that report.

When the available homologous sequences are few, existing methods, including our methods, are incapable of predicting contacts accurately, although our method is effective to some degree for cases of shallow MSAs. As the next step, we would like to improve the MSA construction process and to collect sufficient evolutional information from wider sequence spaces through extensive research.

**Fig. 6:**
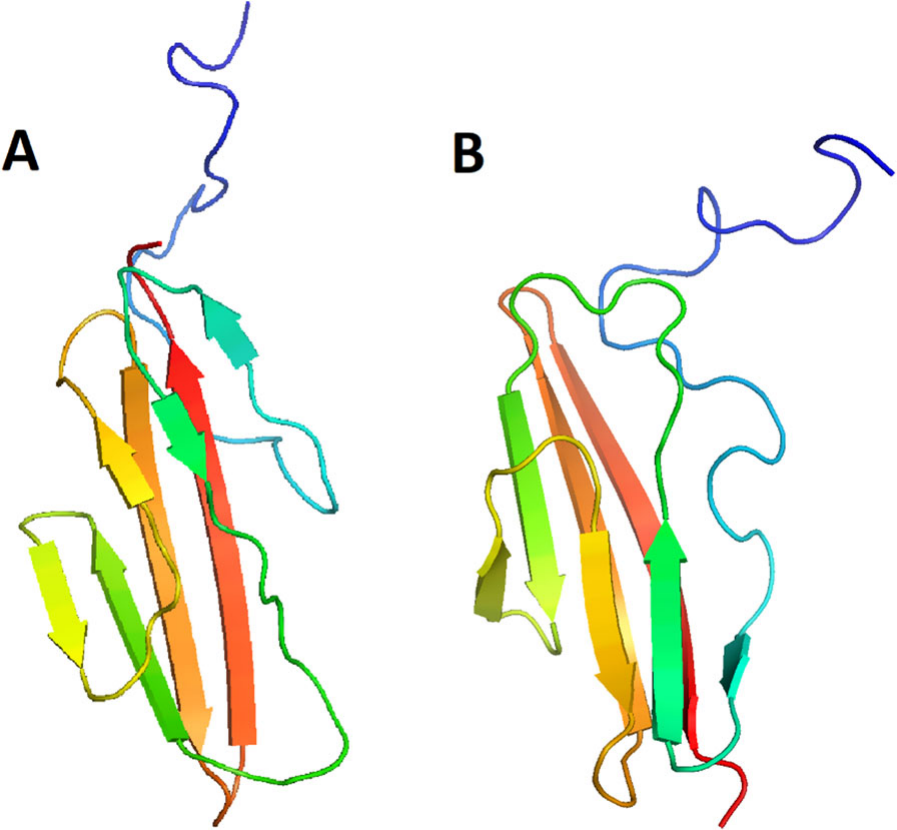
Badly predicted model obtained in spite of good predicted contacts: (**a**) predicted model and (**b**) native structure

As for tertiary structure prediction, some proteins exist for which we cannot obtain good models, even though our contact prediction results are fairly good. One example of these results is T0845-D1. For this protein, the predicted contact accuracy is 86.6% (for top *L* prediction), but the resultant TMscore is 0.276. Figure 6 portrays the structure of this sample. The general shape of this predicted model is similar to the native structure, but all strands go in opposite directions against the native structure. Actually, T0845 is a 97-residue protein with 127 long-range contacts (1.32 L). In this case, 86.6% top *L* prediction is insufficient. More precise contact information would be necessary to solve such a mirror image-like problem. Furthermore, more sophisticated tertiary structure construction methods are necessary.

## Conclusions

As described in this paper, we propose an end-to-end learning framework of protein contact prediction that can effectively use information derived from either deep or shallow MSAs. For deep MSAs, our model can perform weighting of the sequences in MSA to eliminate noise sequences and to gain accuracy. However, for shallow MSAs, it is useful to add some features derived from the sequence itself and MSA to improve the accuracy. Results demonstrate that our model can obtain good results compared with existing ECA methods such as PSICOV, CCMpred, DeepCOV, and ResPRE when tested on the CASP11 and CASP12 datasets. Moreover, our Multi-task Model is good at predicting secondary structures. Using these predicted contact and secondary structures, we can obtain more accurate three-dimensional models of a target protein than those obtained using existing ECA methods, starting from its MSA.

## Method

### Datasets

An original dataset was prepared for this study using the following steps. 1) A set of non-redundant amino acid sequences was obtained from PISCES, a PDB sequence culling server (30% sequence identity cutoff, 2.5 Å resolution cutoff, 1.0 R-factor cutoff, 15,209 total number of chains as of April 5, 2018) [30]. 2) PDB files were retrieved. Then true contact pairs were calculated from the protein coordinates. For this study, we defined a contact if the distance of C_β_ atoms of the residue pair was less than 8 Å. For glycine residues, C_α_ atoms were used instead of C_β_ atoms. The PDB coordinates include many missing values (in our dataset, more than 5000 proteins have at least one missing value for C_β_ atoms). Therefore, we marked a residue pair that had a missing C_β_ coordinate as NaN and excluded it when we calculated the loss. 3) Removal of redundancy was performed with the test set (see below). We excluded from our dataset those proteins sharing > 25% sequence identity or having a BLAST *E*-value < 0.1 with any test protein by blastp [31]. 4) Proteins with length greater than 700 residues or with fewer than 25 residues were also eliminated. At this stage, our dataset comprised 13,262 protein chains. In ensemble averaging (see below), we split them into five (up to ten) sets and used one of them as a validation set. We used the remaining sets as training sets for the respective models. For our Multi-task Model described below, secondary structures and solvent-accessible surface areas of proteins were calculated using DSSP [32]. We used only those proteins for which the secondary structure states could be assigned for 80% or more of their residues. We noticed that one protein, 12AS had been removed by error. Consequently, 1938 protein chains were excluded from the 13,262 protein chains. For fair comparison between our models, the remaining 11,324 protein chains were used in all experiments. We used one of our five training/validation datasets to evaluate effects of weighting sequences in an MSA (results shown in Tables 2 and 3 and Fig. 3). This dataset includes 9058 protein chains for training and 2266 protein chains for validation. As the test sets for benchmarking our methods, we used the CASP11 (105 domains) and CASP12 (55 domains) dataset [33, 34] obtained from the CASP download area (http://www.predictioncenter.org/download_area/). We prepared MSAs for proteins in both our original and test datasets using HHblits [35] with three iterations. The threshold *E*-value was set to 0.001 on the UniProt20_2016 library. Sequence coverage was set to 60% using the “-cov” option. These settings were the same as those used in PSICOV.

### Neural network models

We developed our neural network models to achieve improvement in the respective precisions of both shallow and deep MSAs. Moreover, we expanded our model to a multi-task model to increase the prediction accuracy by incorporation with predictions of secondary structures and solvent-accessible surface areas. Methods using convolutional neural networks (CNNs), which are widely applied to image classification tasks, have been used successfully for protein contact prediction [36]. Therefore, we also used CNNs in our models.

As in Graphical Lasso methods, our models take covariance matrices calculated from MSAs as their inputs to calculate the probability of contact for each residue pair in a protein. To calculate covariance matrices, we used a formula used for a study of PSICOV, as shown below.

1 $$ S{a}_i{b}_j=f\left({a}_i{b}_j\right)-f\left({a}_i\right)f\left({b}_j\right) $$

**Fig. 7:**
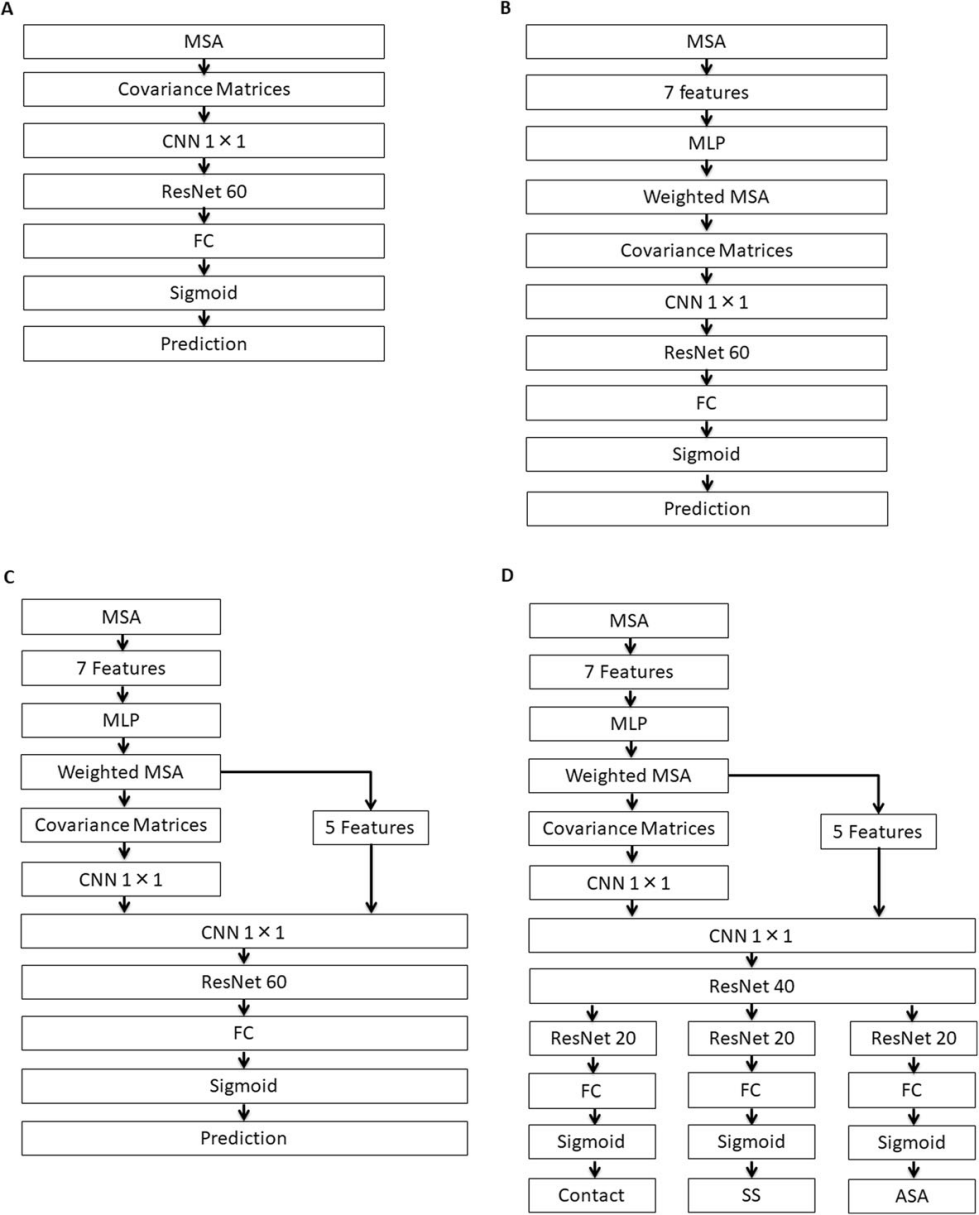
Architectures of the proposed networks: (**a**) Baseline Model, (**b**) Weighted MSA Model, (**c**) Feature Added Model, and (**d**) Multi-task Model

Therein, *a* and *b* respectively represent amino acid types at positions *i* and *j*. Also, *f* (*a*_*i*_) (and *f* (*b*_*j*_)), respectively denote frequencies of amino acid *a* (and *b*) at position *i* (and *j*); *f* (*a*_*i*_*b*_*j*_) stands for the frequency of amino acid pairs *a* and *b* at positions *i* and *j*. If no correlation is found between *i* and *j* with respect to amino acid pairs *a* and *b*, then *Sa*_*i*_*b*_*j*_ is equal to zero. Using this formula with pairs of 21 amino acid type (including a gap), one can obtain 441 *L* × *L* covariance matrices, where *L* signifies the sequence length of a target protein. Our input covariance matrices are *L* × *L* pixel images with 441 channels: typical color images have three channels. Therefore, we can apply a CNN. For this study, we adopt a residual network [37] to deepen the model and to achieve higher accuracy. We tested the four model variants described below. Their architectures are presented in Fig. 7.

A) Baseline Model: First, in this model, 441 channels of *L* × *L* covariance matrices calculated from MSAs are fed into a 1 × 1 CNN to reduce the dimensionality of channels to 128. Then the matrices are fed into the 30-block residual network. Each residual block has two CNN layers. The total number of layers in our residual network is 60. We used 60 layers because of GPU memory limitations. Each output of the residual network is 128 channels of *L* × *L* matrices. We transform them and feed them into a fully connected layer and sigmoid function to obtain contact probabilities.

B) Weighted MSA Model: To reduce noise of MSA, we weight each sequence of an MSA in this model. This weighting is also assigned using a neural network. First, we use a multilayer perceptron (MLP) network to calculate the weight for each sequence in an MSA using features of seven types: the number of sequences in an MSA, sequence identity with a target sequence, sequence identity with a consensus sequence of an MSA, the gap ratio for each sequence, and average values of the last three features (i.e., sequence identities and a gap ratio). The MLP, which has two hidden layers and for which each hidden layer has seven nodes, are used for this task. The output of this network is then used to weight each sequence in an MSA. Subsequently, based on the weighted MSA, 441 *L* × *L* covariance matrices are calculated and are fed into a 1 × 1 CNN. Because all these calculations can be written as matrix operations and because they can be represented by one connected network, gradients of loss function with respect to each variable in MLP and CNN are calculable through backpropagation. Consequently, the network can be optimized completely in an end-to-end manner.

C) Feature Added Model: To this model, we add five features: a query sequence, a Position Specific Score Matrix (PSSM), entropy of each column of weighted MSA, mutual information of each column pair of weighted MSA, and sequence separations calculated from query sequences. The first three features are 1D features of length *L*. These 1D features are stacked *L* times vertically to shape *L* × *L* matrices. We also used a transposed version of these matrices because information of both *i* and *j* at position (*i*, *j*) must be obtained. We treat query sequences and PSSMs as categorical variables and apply one-hot encoding to these features. The final dimensions of these features are (*L*, *L*, 20 × 2) for query sequences, (*L*, *L*, 21 × 2) for PSSMs, and (*L*, *L*, 1 × 2) for entropy. The final dimensions of both mutual information and sequence separations are (*L*, *L*, 1). Finally, after concatenating these features to covariance matrices and reducing their dimensionality to 128, we feed them into residual networks.

D) Multi-task Model: Secondary structures are also key elements to predict tertiary structures. Multi-task learning, a common technique of DNN [38, 39] is also used in protein research [40]. In our case, we try to predict contacts, secondary structures, and accessible surface areas simultaneously using multi-task learning. Although the network is based on the Feature Added model, after 20 blocks of residual network, we separate the residual blocks for each task: we share the parameters of 20 residual blocks within these three tasks and do not share the last 10 residual blocks. Finally, the outputs of these residual blocks are fed respectively into a fully connected layer to predict contacts, secondary structures, and accessible surface areas. For the secondary structures and accessible surface areas, we use an *i*-th row and an *i*-th column of the *L* × *L* matrices and concatenate them as features of *i*-th residues.

We calculate the losses separately and add them for joint training.

Total Loss = Loss Contact + Loss Secondary Structure + Loss Accessible Surface Area (2).

We define each term, in eq. (2), as

3 $$ \mathrm{Contact}\kern0.28em \mathrm{Loss}=-{\sum}_{ij}\left({y}_{Contact\kern0.28em ij}\log {p}_{Contact\kern0.28em ij}+\left(1-{y}_{Contact\kern0.28em ij}\right)\log \left(1-{P}_{Contact\kern0.28em ij}\right)\right) $$

where *y*_*contact ij*_ is the true label (1 for contact, otherwise 0) for the residue pair of (*i*, *j*) positions and *p*_*contact ij*_ is the predicted contact probability. The summation is calculated over all residue pairs of (*i*, *j*), except when the true label is not missing values.

4 $$ \mathrm{Secondary}\kern0.28em \mathrm{Structure}\kern0.28em \mathrm{Loss}=-{\sum}_k\left({y}_{Helix\kern0.28em k}\log {p}_{Helix\kern0.28em k}+{y}_{Sheet\kern0.28em k}\log {p}_{Sheet\kern0.28em k}+{y}_{Coil\kern0.28em k}\log {p}_{Coil\kern0.28em k}\right) $$

Therein, *y*_*Helix k*_, *y*_*Sheet k*_, and *y*_*Coil k*_ respectively represent the one-hot encoded true label for the *k*_*th*_ residue of helix, sheet, and coil. In addition, *p*_*Helix k*_, *p*_*Sheet k*_, and *p*_*Coil k*_ respectively denote their predicted probabilities. The summation is calculated over all residues, except when the true label is missing.

5 $$ \mathrm{Accessible}\ \mathrm{Surface}\ \mathrm{Area}\ \mathrm{Loss}=\sqrt{\frac{\sum_k{\left( AS{A}_{true\kern0.24em k}- AS{A}_{pred\;k}\right)}^2}{N}} $$

In that equation, *ASA*_*true k*_ and *ASA*_*pred k*_ respectively stand for the accessible surface area of the true value and the predicted value of the *k*_*th*_ residue. In addition, *N* signifies the total number of residues calculated from the accessible surface area. The summation is over the same residues as those used in the case of secondary structures.

For our experiments, all filter sizes of convolutional operations in the residual network are 3 × 3. The ReLU activation function is used. We trained all these networks using the ADAM optimizer with the learning rate of 0.0005. Batch normalization is used to obtain higher accuracy and faster convergence. One batch includes the data of one domain. Proteins have their different lengths. Therefore, input matrices can have different sizes. However, because the number of our network parameters is independent of protein length, we can deal comprehensively with proteins of different lengths. Furthermore, by calculating the gradient and updating the network parameters by one batch size, we obviate the use of zero padding. All hyperparameters and network architectures such as the number of layers and variation of connections are selected according to the results achieved for validation sets. All experiments were conducted using an ordinary desktop computer with a GPU (GeForce TITAN X; Nvidia Corp.) using the TensorFlow library. Training required several days to calculate 20–30 epochs.

### Ensemble averaging

To raise accuracy, we used ensemble averaging. We split our dataset into five sets. Consequently, we were able to obtain five (or up to ten) different models trained with five (or up to ten; see Table 10) different sets. Our final prediction result for each residue pair was obtained simply by averaging these predicted probabilities.

### Cropping and sampling

To overcome the GPU memory size limitation and to deepen the network, we crop a part of the protein sequences and sample the sequences in MSAs. More concretely, when the sequence length is greater than 200 residues, we crop 200 residues from all protein sequences. When the number of sequences in MSAs is greater than 30,000, we sample 30,000 sequences from them. That number is adequate because our residual network has 3 × 3 filters and 60 layers and because it covers only 121 × 121 of the covariance matrices. We observed decreased prediction accuracy for sampling numbers less than 10,000. These cropping and sampling are only done during training. Entire sequences and MSAs are used during prediction.

### Evaluation of prediction results

To assess contact prediction accuracies, we compared our results with those obtained using existing prediction methods. According to sequence separations of residue pairs, we defined the contact types as “short” 6 < =|*i* - *j*| < =11, “medium” 12 < =|*i* - *j*| < =23, and “long” 24 < =|*i* - *j*|, and compared the top *L*/*k* (*k* = 10,5,2,1) prediction results as described by Wang et al. [19]. The prediction accuracy (precision) was calculated using the following eq.

TP / (TP + FP) (6).

In that equation, TP represents the number of true contacts among the predicted ones: TP + FP is the number of all predicted contacts. We selected PSICOV, CCMpred, DeepCov and ResPRE as representatives of ECA methods and selected MetaPSICOV, DeepMetaPSICOV and RaptorX-Contact as representatives of meta-predictors to be compared. We performed calculations with our own local prediction directed by instructions for using each method. The same MSAs used in our models are also used for these models except for MetaPSICOV and RaptorX-Contact. For MetaPSICOV “–id 99” option was used in its default setting. For the RaptorX-Contact, no local execution file was available. Predictions were calculated on their server. However, for 3 out of 105 CASP11 domains and for 1 out of 55 CASP12 domains, the results were not retrieved because of server error. The MSAs were prepared by their server originally. They differed from ours. Using the CASP11 and CASP12 datasets, we calculated the accuracy for each separate domain, not an entire protein.

For evaluation of secondary structure and for accessible surface area prediction, we used RaptorX-Property and SCRATCH-1D as state-of-the-art methods. We calculated the results obtained using local prediction. To evaluate prediction results of secondary structure, we also measured recall: TP/(TP + FN).

### Tertiary structure prediction

To predict tertiary structures from obtained contacts and secondary structure predictions, we used a script in the CONFOLD package. We mixed up all three (short, medium, and long) ranges of predicted contacts, ordered them by their probability of contact; then we used (up to) the top 2 *L* contacts among them as inputs for the script.

## Supplementary information

**Additional file 1: Figure S1.** Distributions of weight values of gap ratio, sequence identity and sequence identity with a consensus sequence. Each dot represents a sequence in each MSA. These protein domains (1JF3A, 2R6UA and 2RDEA) are randomly selected from on validation dataset.

## Data Availability

https://github.com/tomiilab/DeepECA

## Abbreviations

CASP: Critical assessment of protein structure prediction
CNN: Convolutional neural network
DNN: Deep neural network
ECA: Evolutionary coupling analysis
MLPs: Multilayer perceptrons
MSA: Multiple sequence alignment
PSSM: Position specific score matrix

## References

[1] Dunn SD, Wahl LM, Gloor GB (2008). Mutual information without the influence of phylogeny or entropy dramatically improves residue contact prediction. Bioinformatics.

[2] Björkholm P, Daniluk P, Kryshtafovych A, Fidelis K, Andersson R, Hvidsten TR (2009). Using multi-data hidden Markov models trained on local neighborhoods of protein structure to predict residue-residue contacts. Bioinformatics.

[3] Balakrishnan S, Kamisetty H, Carbonell JG, Lee SI, Langmead CJ (2011). Learning generative models for protein fold families. Proteins.

[4] Jones DT, Buchan DW, Cozzetto D, Pontil M (2012). PSICOV: precise structural contact prediction using sparse inverse covariance estimation on large multiple sequence alignments. Bioinformatics.

[5] Marks DS, Colwell LJ, Sheridan R, Hopf TA, Pagnani A, Zecchina R, Sander C (2011). Protein 3D structure computed from evolutionary sequence variation. PLoS One.

[6] Di Lena P, Nagata K, Baldi P (2012). Deep architectures for protein contact map prediction. Bioinformatics.

[7] Kamisetty H, Ovchinnikov S, Baker D (2013). Assessing the utility of coevolution-based residue-residue contact predictions in a sequence- and structure-rich era. Proc Natl Acad Sci U S A.

[8] Eickholt J, Cheng J (2013). A study and benchmark of DNcon: a method for protein residue-residue contact prediction using deep networks. BMC Bioinformatics.

[9] Wang Z, Xu J (2013). Predicting protein contact map using evolutionary and physical constraints by integer programming. Bioinformatics.

[10] Ekeberg M, Lovkvist C, Lan Y, Weigt M, Aurell E (2013). Improved contact prediction in proteins: using pseudolikelihoods to infer Potts models. Phys Rev E Stat Nonlinear Soft Matter Phys.

[11] Ekeberg M, Hartonen T, Aurell E (2014). Fast pseudolikelihood maximization for direct-coupling analysis of protein structure from many homologous amino-acid sequences. J Comput Phys.

[12] Kaján L, Hopf TA, Kalaš M, Marks DS, Rost B (2014). FreeContact: fast and free software for protein contact prediction from residue co-evolution. BMC Bioinformatics.

[13] Seemayer S, Gruber M, Söding J (2014). CCMpred – fast and precise prediction of protein residue-residue contacts from correlated mutations. Bioinformatics.

[14] Jones DT, Singh T, Kosciolek T, Tetchner S (2015). MetaPSICOV: combining coevolution methods for accurate prediction of contacts and long range hydrogen bonding in proteins. Bioinformatics.

[15] Andreani J, Söding J (2015). bbcontacts: prediction of b-strand pairing from direct coupling patterns. Bioinformatics.

[16] Ma J, Wang S, Wang Z, Xu J (2015). Protein contact prediction by integrating joint evolutionary coupling analysis and supervised learning. Bioinformatics.

[17] Li Q, Dahl DB, Vannucci M, Joo H, Tsai JW (2016). KScons: a Bayesian approach for protein residue contact prediction using the knob-socket model of protein tertiary structure. Bioinformatics.

[18] Golkov V, Skwark MJ, Golkov A, Dosovitskiy A, Brox T, Meiler J, Cremers D. Protein contact prediction from amino acid co-evolution using convolutional networks for graph-valued images. NIPS Proceedings. 2016.

[19] Wang S, Sun S, Li Z, Zhang R, Xu J (2017). Accurate De novo prediction of protein contact map by ultra-deep learning model. PLoS Comput Biol.

[20] Friedman J, Hastie T, Tibshirani R (2008). Sparse inverse covariance estimation with the graphical lasso. Biostatistics.

[21] Jones DT, Kandathil SM (2018). High precision in protein contact prediction using fully convolutional neural networks and minimal sequence features. Bioinformatics.

[22] Li Yang, Hu Jun, Zhang Chengxin, Yu Dong-Jun, Zhang Yang (2019). ResPRE: high-accuracy protein contact prediction by coupling precision matrix with deep residual neural networks. Bioinformatics.

[23] Kandathil Shaun M., Greener Joe G., Jones David T. (2019). Prediction of interresidue contacts with DeepMetaPSICOV in CASP13. Proteins: Structure, Function, and Bioinformatics.

[24] Fox G, Sievers F, Higgins DG (2016). Using de novo protein structure predictions to measure the quality of very large multiple sequence alignments. Bioinformatics.

[25] Zhang Y, Skolnick J (2004). Scoring function for automated assessment of protein structure template quality. Proteins.

[26] Wang S, Li W, Liu S, Xu J (2016). RaptorX-property: a web server for protein structure property prediction. Nucleic Acids Res.

[27] Magnan CN, Baldi P (2014). SSpro/ACCpro: almost perfect prediction of protein secondary structure and relative solvent accessibility using profiles, machine learning and structural similarity. Bioinformatics.

[28] Adhikari Badri, Bhattacharya Debswapna, Cao Renzhi, Cheng Jianlin (2015). CONFOLD: Residue-residue contact-guidedab initioprotein folding. Proteins: Structure, Function, and Bioinformatics.

[29] Hanson Jack, Paliwal Kuldip, Litfin Thomas, Yang Yuedong, Zhou Yaoqi (2018). Improving prediction of protein secondary structure, backbone angles, solvent accessibility and contact numbers by using predicted contact maps and an ensemble of recurrent and residual convolutional neural networks. Bioinformatics.

[30] Wang G., Dunbrack R. L. (2003). PISCES: a protein sequence culling server. Bioinformatics.

[31] Camacho Christiam, Coulouris George, Avagyan Vahram, Ma Ning, Papadopoulos Jason, Bealer Kevin, Madden Thomas L (2009). BLAST+: architecture and applications. BMC Bioinformatics.

[32] Kabsch Wolfgang, Sander Christian (1983). Dictionary of protein secondary structure: Pattern recognition of hydrogen-bonded and geometrical features. Biopolymers.

[33] Monastyrskyy Bohdan, D'Andrea Daniel, Fidelis Krzysztof, Tramontano Anna, Kryshtafovych Andriy (2015). New encouraging developments in contact prediction: Assessment of the CASP11 results. Proteins: Structure, Function, and Bioinformatics.

[34] Schaarschmidt Joerg, Monastyrskyy Bohdan, Kryshtafovych Andriy, Bonvin Alexandre M.J.J. (2017). Assessment of contact predictions in CASP12: Co-evolution and deep learning coming of age. Proteins: Structure, Function, and Bioinformatics.

[35] Remmert Michael, Biegert Andreas, Hauser Andreas, Söding Johannes (2011). HHblits: lightning-fast iterative protein sequence searching by HMM-HMM alignment. Nature Methods.

[36] Adhikari B. DEEPCON: Protein Contact Prediction using Dilated Convolutional Neural Networks with Dropout. bioRxiv. 2019:590455. 10.1093/bioinformatics/btz593 31359036

[37] He K, Zhang X, Ren S, Sun J. Deep Residual Learning for Image Recognition. In Proceedings of the IEEE Conference on Computer Vision and Pattern Recognition (CVPR), IEEE. 2016;77:770–8.

[38] Caruana Rich (1997). Machine Learning.

[39] Yu Z, Qiang Y. A Survey on Multi-Task Learning. arXiv: 1707.08114 2018.

[40] Heffernan R, Paliwal K, Lyons J, Dehzangi A, Sharma A, Wang J, Sattar A, Yang Y, Zhou Y. Improving prediction of secondary structure, local backbone angles, and solvent accessible surface area of proteins by iterative deep learning. Nat Sci Rep. 2015;5:11476. 10.1038/srep11476 PMC4476419 26098304

