# Supplementary figures and images for: DeepECA: an end-to-end learning framework for protein contact prediction from a multiple sequence alignment
Source: BMC Bioinformatics. 2020 Jan 9;21:10. doi: 10.1186/s12859-019-3190-x (PMC6953294; doi:10.1186/s12859-019-3190-x)

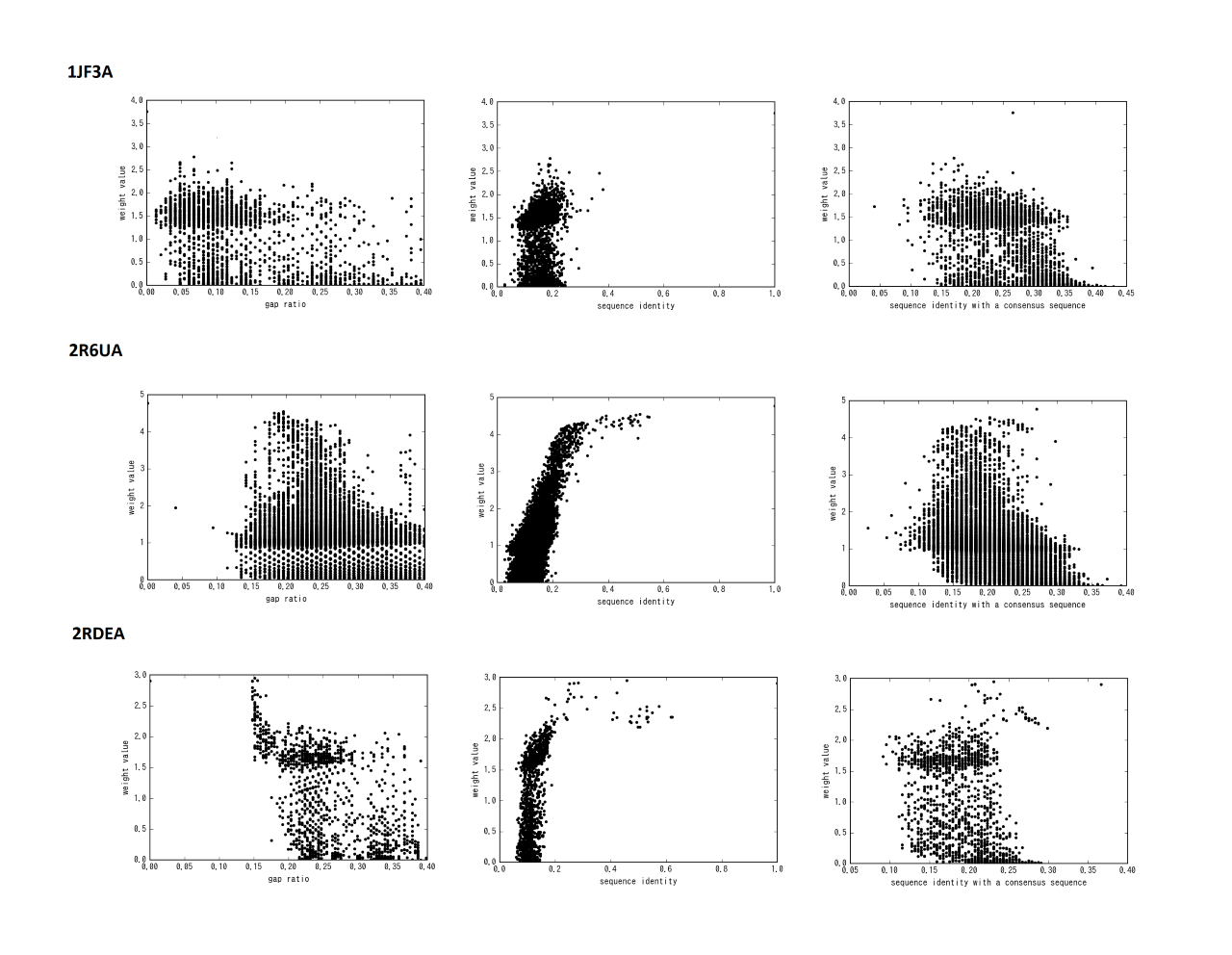

Supplement: Supplementary file 1 — Additional file 1: Figure S1. Distributions of weight values of gap ratio, sequence identity and sequence identity with a consensus sequence. Each dot represents a sequence in each MSA. These protein domains (1JF3A, 2R6UA and 2RDEA) are randomly selected from on validation dataset. [file 12859_2019_3190_MOESM1_ESM.png]
